# Hsa_circ_0062270 Promotes Tumorigenesis of Melanoma by Stabilizing the Linear Transcript Cell Division Cycle Protein 45

**DOI:** 10.3389/fgene.2022.897440

**Published:** 2022-05-10

**Authors:** Cuie Wei, Wentao Sun, Changhai Liu, Fanjun Meng, Lele Sun, Xiangsheng Ding

**Affiliations:** ^1^ Department of Plastic Surgery, The First People’s Hospital of Lianyungang, Lianyungang, China; ^2^ Department of Plastic Surgery, The First Affiliated Hospital of Kunming Medical University, Kunming, China

**Keywords:** hsa_circ_0062270, melanoma, Cdc45, proliferation, metastasis

## Abstract

**Background:** To elucidate the potential biological function of hsa_circ_0062270 in the malignant process of melanoma and its potential target.

**Methods:** Quantitative real-time polymerase chain reaction (qRT-PCR) was conducted to examine relative level of hsa_circ_0062270 in melanoma tissues and normal skin tissues. The diagnostic and prognostic potentials of hsa_circ_0062270 in melanoma were evaluated. The regulatory effect of hsa_circ_0062270 on the expression of linear transcript Cell division cycle protein 45 (CDC45) was also examined.

**Results:** Hsa_circ_0062270 was up-regulated in melanoma samples and cell lines, which displayed certain diagnostic and prognostic potentials in melanoma. Inhibition of hsa_circ_0062270 attenuated the proliferative, migratory and invasive functions. Hsa_circ_0062270 could stabilize the expression of linear transcript CDC45, and thus participated in the malignant process of melanoma.

**Conclusion:** Hsa_circ_0062270 promotes proliferative, migratory and invasive functions of melanoma cells *via* stabilizing the linear transcript CDC45. Hsa_circ_0062270 can be used to diagnosis and treatment of melanoma.

## Introduction

Melanoma is a highly malignant skin cancer derived from melanocytes. Melanoma accounts for more than 70% of skin cancer deaths (Testori et al., 2017). More seriously, estimated numbers of new cases and death cases of melanoma are on the rise. Surgical resection combined lymph node dissection is an effective treatment for early stage melanoma (Chattopadhyay et al., 2016). Nevertheless, most of melanoma patients cannot be operated because of metastasis, leading to a poor 5-year survival (20%) (Shain and Bastian, 2016). Molecular mechanisms underlying melanoma process and metastasis remain largely unclear.

CircRNAs are novel noncoding RNAs to be widely analyzed. They are extensively involved in various fields of life sciences (Hsiao et al., 2017; Chen and Huang, 2018; Han et al., 2018). Since circRNAs do not have 3′ end, 5′ end and poly (A) tail, they can escape degradation from exonucleases. CircRNAs are more conservative and stable than linear RNAs. Moreover, they are evolutionarily conserved in different species, and specifically expressed in different tissues and developmental stages (Dong et al., 2019; Patop et al., 2019). According to the origins, circRNAs are classified to circular exonic RNAs that only contain reverse-splicing exons, circular intronic RNAs (ciRNAs) that only contain reverse-splicing introns, and exon-intron circRNAs (ElciRNAs) that introns are remained in circular exons (Salzman, 2016). The following mechanisms explain the biological functions of circRNAs: 1) CircRNAs inhibit miRNA activities through exerting the miRNA sponge effect; 2) CircRNAs interact with RNA-binding proteins as protein sponges; 3) CircRNAs regulate Pol II transcription of parent genes; 4) CircRNAs regulate linear splicing through competitively targeting splicing sites of pre-mRNAs; 5) CircRNAs have protein-encoding ability and can translate proteins (Kristensen et al., 2019). Abundant evidences have proven the vital functions of circRNAs in physiological and pathological processes (Salzman, 2016; Qu et al., 2017).

Hsa_circ_0062270 is located on chromosome 22: 19496052-19502571, and its gene symbol is CDC45. Evidence has showed that hsa_circ_0062270 is obviously up-regulated in melanoma (Hao et al., 2021). A previous study have demonstrated that downregulation of hsa_circ_0062270 can inhibit the progression of melanoma, however, the mechanism still remains unclear (Hao et al., 2021). The aim of the current research was to explore the biological effect of hsa_circ_0062270 on malignant phenotypes of melanoma and its potential target.

## Patients and Methods

### Subjects and Specimens

The normal skin tissues and melanoma tissues of 50 patients with melanoma in our hospital were selected. The ethics committee of The First People’s Hospital of Lianyungang approved our study. Signed written informed consents were obtained from all participants before the study.

### Cell Culture

Melanoma cells (SKMEL1, A375, A2058 and A875) and normal human epidermal melanocytes (NHEM) were provided by Cell Bank of Type Culture Collection (Shanghai, China). Cells were cultivated in DMEM containing 10% fetal bovine serum (FBS), 100 U/mL penicillin and 100 μg/ml streptomycin at 5% CO_2_, 37°C. Cell transfection was performed using Lipofectamine 3,000 as per the protocols. Cell proliferation was determined by EdU (Beyotime, Shanghai, China).

### Quantitative Real-Time Polymerase Chain Reaction

RNAs isolation was done with TRIzol and were then reversely transcribed into cDNAs. U6 and GAPDH were used as the internal controls with the method of 2^−ΔΔCt^. Primers used were shown below: hsa_circ_0062270: Forward: 5′-AGG​ATG​GCT​CAG​GGA​CAG​AT-3′, reverse: 5′-AGG​CCA​TGG​TAC​AGC​TTG​TC-3′; CDC45: Forward: 5′-TTC​GTG​TCC​GAT​TTC​CGC​AAA-3′, reverse: 5′-TGG​AAC​CAG​CGT​ATA​TTG​CAC-3′; GAPDH: Forward: 5′-CGG​AGT​CAA​CGG​ATT​TGG​TCG​TAT-3′, reverse: 5′-AGC​CTT​CTC​CAT​GGT​GGT​GAA​GAC-3′: U6: Forward: 5′-GCT​GAG​GTG​ACG​GTC​TCA​AA-3′, reverse: 5′-GCC​TCC​CAG​TTT​CAT​GGA​CA-3′.

### Actinomycin D and Rnase R Assays

A375 cells were exposed to Actinomycin D (3 μg/ml). They were collected for isolating total RNAs. Expressions of hsa_circ_0062270 and CDC45 were detected by Quantitative real-time polymerase chain reaction. Cellular RNA (4 mg) was treated either with RNase R (10 U/μg) at 37°C for 30 min or not, followed by purification using RNeasy MinElute (Qiagen, Hilden, Germany).

### Cell Transwell Assay

Cells were seeded into the top chamber and bottom chamber. After 48-h incubation, cells in the bottom were fixed, dyed in crystal violet and captured. Migratory cells were counted in five randomly selected fields per sample. Invasion assay was conducted using transwell chamber precoated with 100 μL of Matrigel (Corning, Corning, NY, United States). In detail, Matrigel was diluted in serum-free medium at 1:3, which was coated on the top of a chamber.

### Statistical Analysis

Data were expressed as mean ± SD (standard deviation) and they were processed using Statistical Product and Service Solutions (SPSS) 20.0 (IBM, Armonk, NY, United States). Prognostic value of hsa_circ_0062270 in melanoma were evaluated by Kaplan-Meier and receiver operating characteristic (ROC) method, respectively. The correlation between hsa_circ_0062270 and CDC45 levels was assessed through Pearson correlation test. A significant difference was set at *p* < 0.05.

## Results

### Up-Regulated hsa_circ_0062270 Predicted Poor Prognosis of Melanoma

**FIGURE 1:**
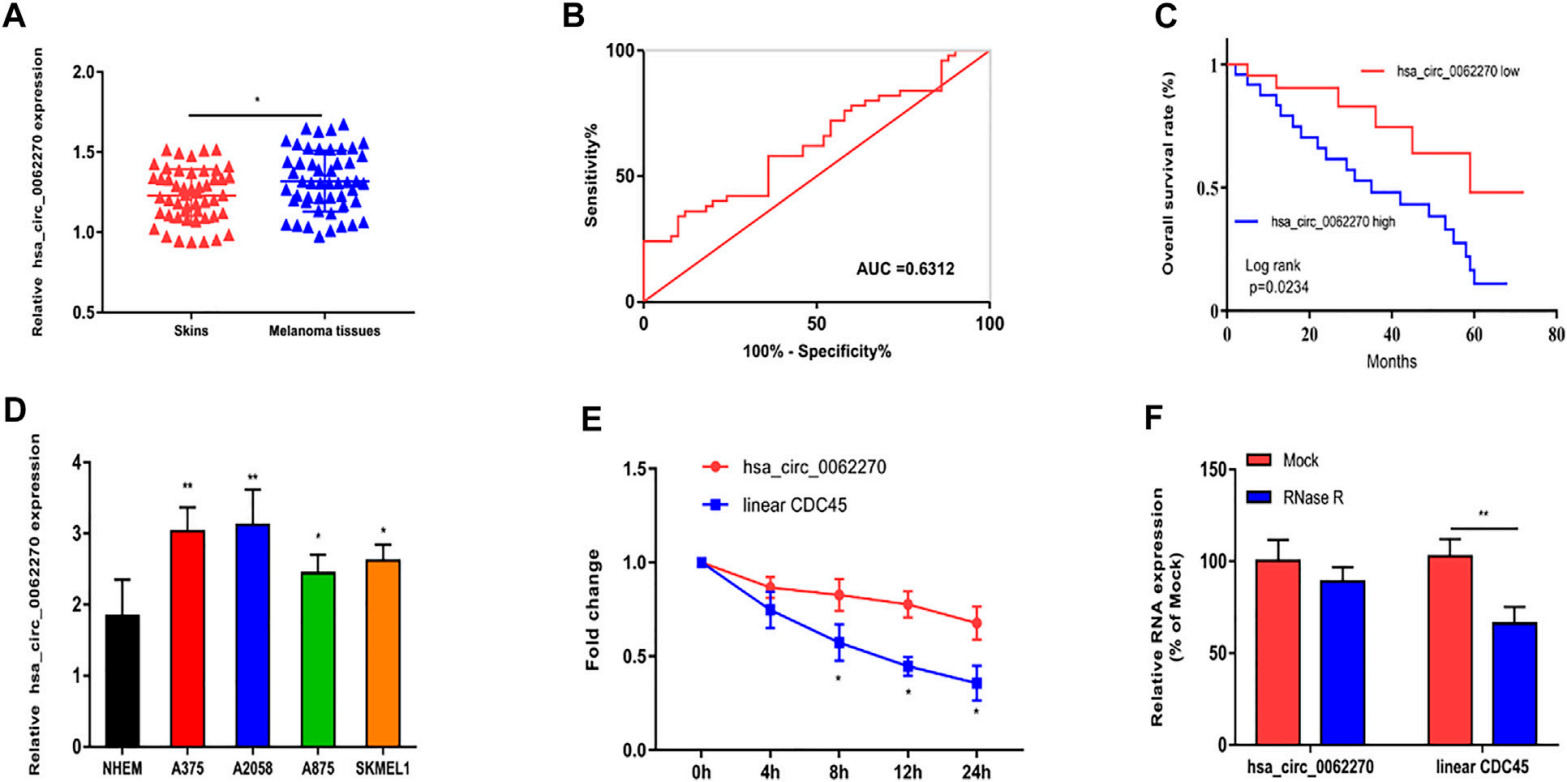
Up-regulated hsa_circ_0062270 predicted poor prognosis in melanoma. **(A)** Expression of hsa_circ_0062270 in melanoma and normal skin tissues; **(B)** Diagnostic potential of hsa_circ_0062270 in melanoma; **(C)** Prognostic potential of hsa_circ_0062270 in melanoma; **(D)** Differential level of hsa_circ_0062270 in melanoma cells and normal human epidermal melanocytes; **(E)** Expression changes of hsa_circ_0062270 and CDC45 in A375 cells treated with Actinomycin D; **(F)** Half-life of hsa_circ_0062270 and CDC45 in A375 cells treated with RNase R. *p < 0.05; **p < 0.01.

Firstly, we explored the expression of hsa_circ_0062270 in melanoma tissues and the normal skin tissues. Results revealed that the expression hsa_circ_0062270 in melanoma tissues was significantly higher than that in normal ones (Figure 1A). Then, receiver operating characteristic (ROC) curves depicted that the AUC of hsa_circ_0062270 was 0.6312 (Figure 1B). Kaplan-Meier analysis found that highly expressed hsa_circ_0062270 predicted a poor prognosis for melanoma patients (*p* = 0.0234) (Figure 1C). Hsa_circ_0062270 significantly increased in melanoma cell lines as well (Figure 1D). We furthermore examined the stability of hsa_circ_0062270 by detecting mRNA levels of hsa_circ_0062270 and CDC45 in Actinomycin D-treated A375 cells. Compared with that of CDC45 (<12 h), the half-life of hsa_circ_0062270 was over 24 h (Figure 1E). RNase R induction did not affect expression level of hsa_circ_0062270, but markedly down-regulated CDC54 (Figure 1F). Therefore, we have verified that hsa_circ_0062270 was highly stable in melanoma cells.

### Hsa_circ_0062270 Promoted Melanoma to Proliferate, Migrate and Invade

**FIGURE 2:**
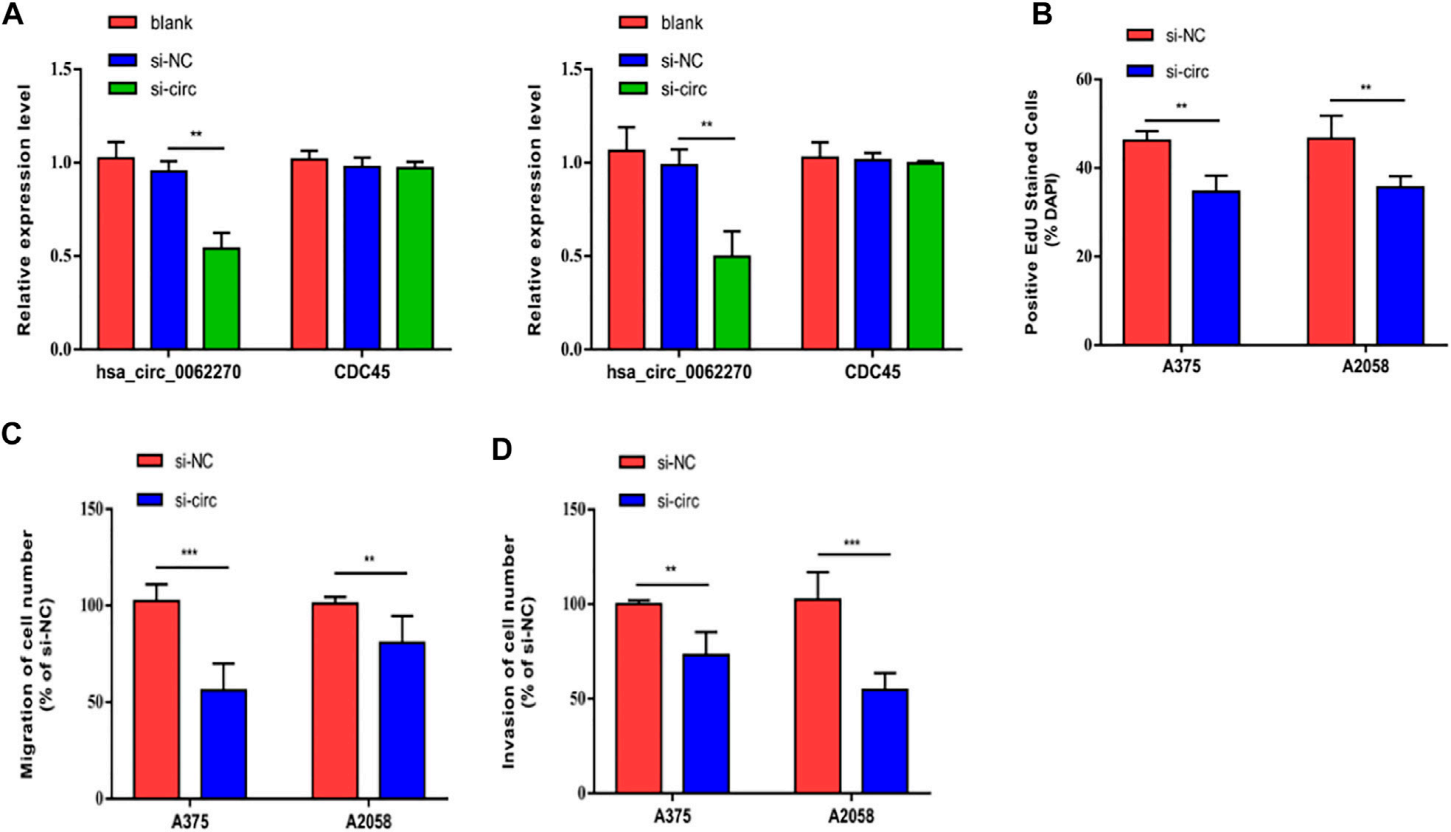
Hsa_circ_0062270 accelerated melanoma to proliferate, migrate and invade. **(A)** Transfection efficacy of hsa_circ_0062270 siRNA; **(B)** EdU-positive rate with hsa_circ_0062270 knockdown; **(C)** Migration in cells with hsa_circ_0062270 knockdown; **(D)** Invasion in cells with hsa_circ_0062270 knockdown. **p < 0.01; ***p < 0.001.

To investigate the effects of hsa_circ_0062270 on melanoma cell proliferation, migration and invasion, cells were treated with hsa_circ_0062270 siRNA. Results indicated that transfection of hsa_circ_0062270 siRNA markedly down-regulated hsa_circ_0062270 level in A375 and A2058 cells (Figure 2A). Knockdown of hsa_circ_0062270 reduced EdU-positive rate in melanoma cells (Figure 2B). In addition, hsa_circ_0062270 siRNA markedly reduced migratory and invasive rates (Figures 2C,D).

### Hsa_circ_0062270 Stabilized CDC45 Expression

**FIGURE 3:**
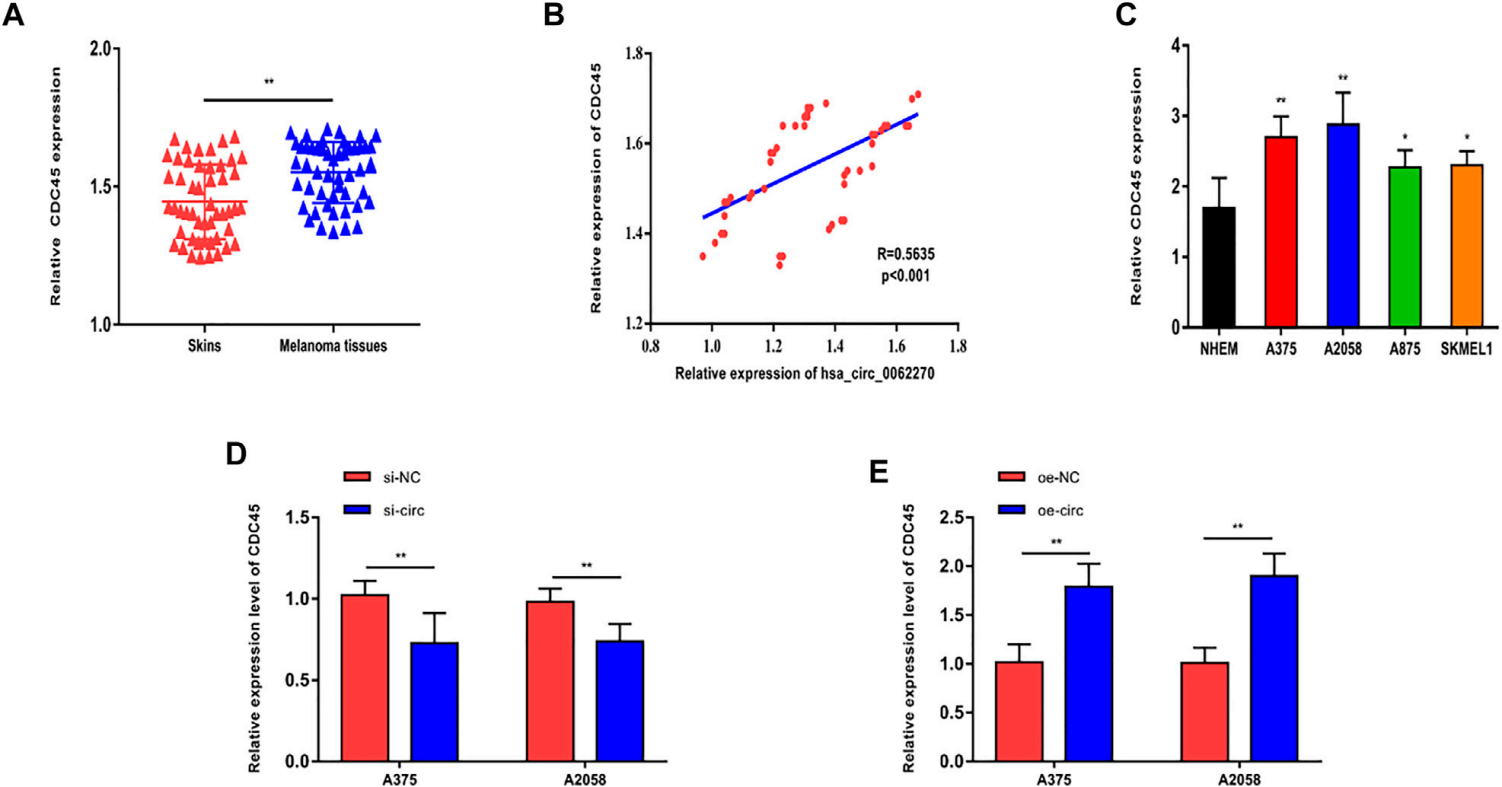
hsa_circ_0062270 stabilized CDC45 expression. **(A)** Differential level of CDC45 in melanoma and normal skin tissues; **(B)** A positive correlation between hsa_circ_0062270 and CDC45 levels in melanoma tissues; **(C)** Differential level of CDC45 in melanoma cells and normal human epidermal melanocytes; **(D)** CDC45 in cells with hsa_circ_0062270 knockdown; **(E)** CDC45 in cells overexpressing hsa_circ_0062270. *p < 0.05; **p < 0.01.

Then we focused on the potential target of hsa_circ_0062270 in the regulation of phenotypes of melanoma. CircRNAs are involved in pathological process *via* mediating expression levels of their linear transcripts. CDC45 was the linear transcript of hsa_circ_0062270 (Figure 3A) and positively correlated to hsa_circ_0062270 level (Figure 3B). Identically, CDC45 was highly expressed in melanoma cell lines (Figure 3C). Knockdown of hsa_circ_0062270 could downregulate CDC45 and as expected, CDC45 was up-regulated in A375 and A2058 cells overexpressing hsa_circ_0062270 (Figures 3D,E). It is concluded that hsa_circ_0062270 could stabilize the expression of CDC45.

### CDC45 Promoted Melanoma to Proliferate, Migrate and Invade

**FIGURE 4:**
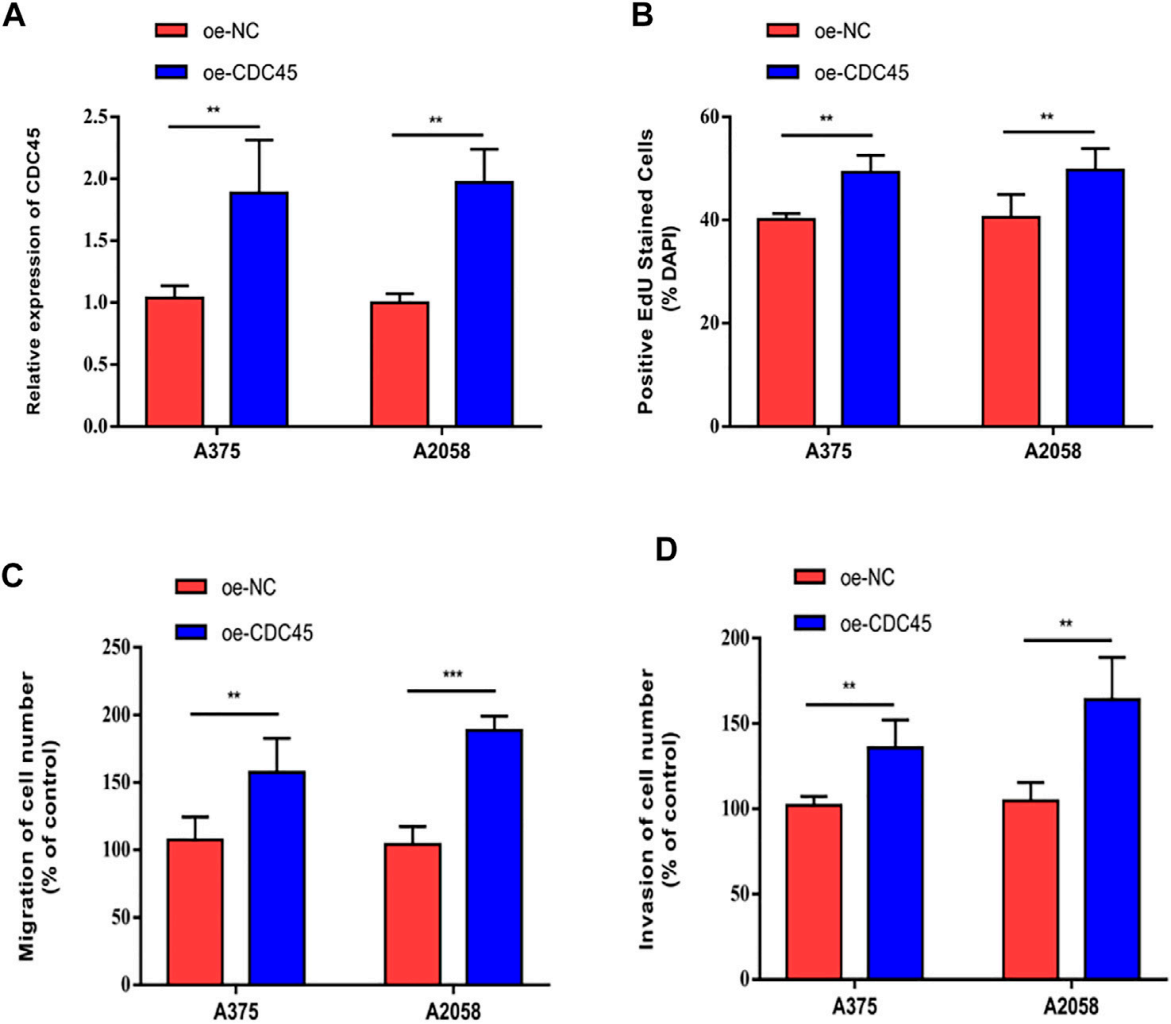
CDC45 promoted melanoma to proliferate, migrate and invade. **(A)** Transfection efficacy of overexpressed plasmid of CDC45; **(B)** EdU-positive rate in cells overexpressing CDC45; **(C)** Migration in cells overexpressing CDC45; **(D)** Invasion in cells overexpressing CDC45. **p < 0.01; ***p < 0.001.

To further elucidate the effects of CDC45 on melanoma phenotypes, we established the overexpression models of CDC45. Transfection of overexpressed plasmid of CDC45 effectively up-regulated CDC45 in A375 and A2058 cells (Figure 4A). In melanoma cells overexpressing CDC45, EdU-positive rate increased (Figure 4B). Moreover, migratory and invasive potentials of melanoma were promoted by overexpressed CDC45 (Figures 4C,D). Therefore, CDC45 could stimulate the malignant process of melanoma.

### Hsa_circ_0062270 Stimulated the Malignant Process of Melanoma by Stabilizing CDC45

**FIGURE 5:**
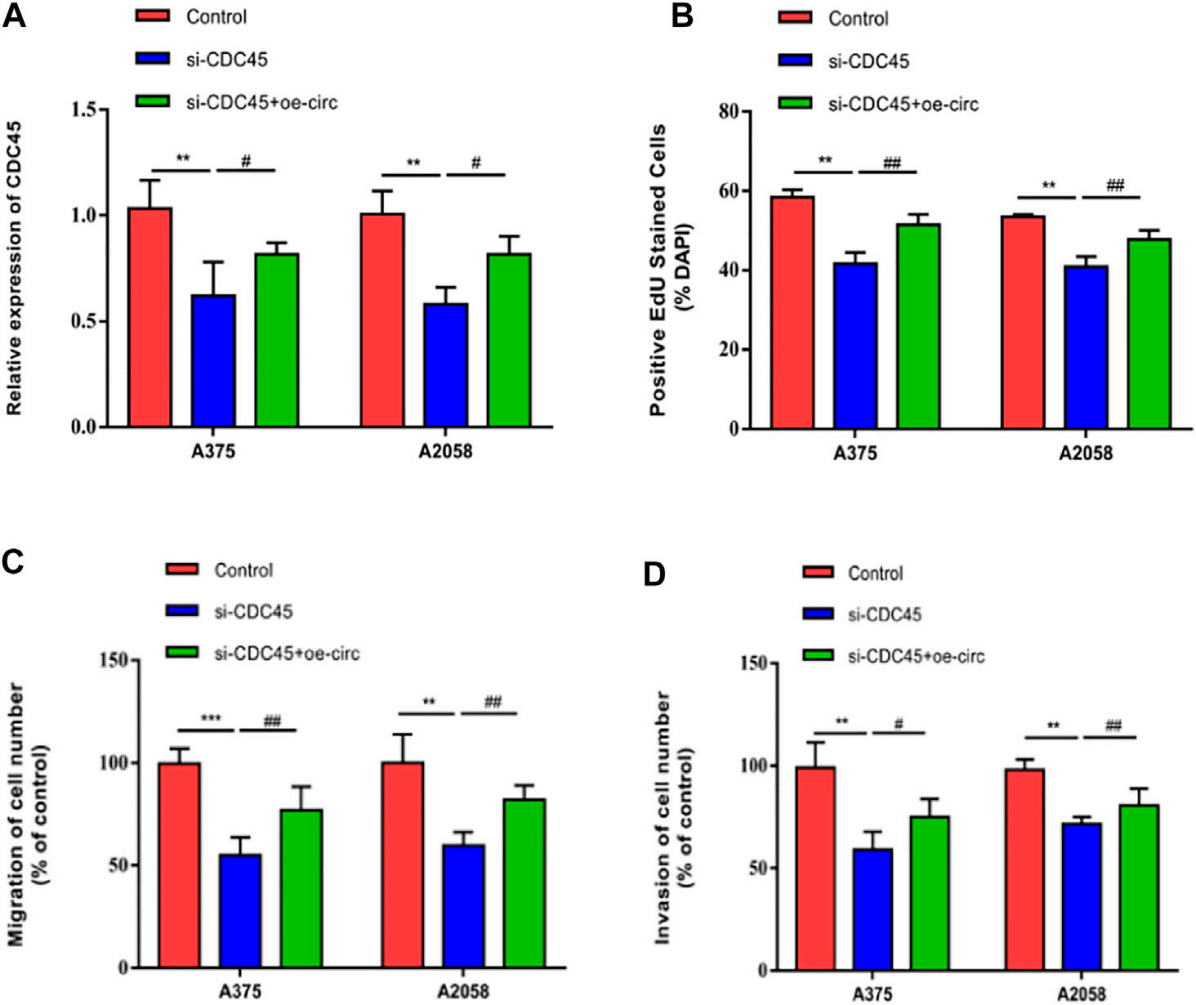
hsa_circ_0062270 stimulated the malignant process of melanoma by stabilizing CDC45. **(A)** Co-transfection of si-CDC34 and overexpressed plasmid of hsa_circ_0062270; **(B)** EdU-positive rate co-regulated by hsa_circ_0062270 and CDC45; **(C)** Migrative ability regulated by hsa_circ_0062270 and CDC45; **(D)** Invasion in A375 and A2058 cells co-regulated by hsa_circ_0062270 and CDC45. **p < 0.01; ***p < 0.001; #*p* < 0.05; ##*p* < 0.01.

To uncover the co-regulation of hsa_circ_0062270 and CDC45, cells were co-transfected using si-CDC45 and overexpressed plasmid of hsa_circ_0062270 (Figure 5A). Overexpression of hsa_circ_0062270 could enhance the down-regulated CDC45 in melanoma cells transfected with si-CDC45. Knockdown of CDC45 reduced EdU-positive rate, migratory cell number and invasive cell number, which were reversed by overexpressed hsa_circ_0062270 (Figures 5B–D). All above indicated that hsa_circ_0062270 may play a carcinogenic role in melanoma by stabilizing its linear transcription CDC45.

## Discussion

The incidence of melanoma is not high, covering 4–5% of malignant tumors. Family history, multiple atypical moles and dysplastic moles are risk factors that trigger the carcinogenesis of melanoma (Pavri et al., 2016). In addition, ultraviolet rays can induce melanoma by damaging DNA repair genes.

CircRNAs, as a type of emerging noncoding RNAs, have been well concerned because of their unique structure and vital functions (Meng et al., 2017; Chen and Huang, 2018). They can be utilized as molecular biomarkers for diagnosing tumors and evaluating their prognosis (Bian et al., 2018; Zhou et al., 2018; Chen et al., 2020). The involvement of circRNAs in melanoma has been reported through literature review.

We previously found that the expression of hsa_circ_0062270 in melanoma was up-regulated. The diagnostic and prognostic potentials of hsa_circ_0062270 in melanoma were verified through depicting ROC and Kaplan-Meier curves, respectively. *In vitro* experiment results illustrated that knockdown of hsa_circ_0062270 remarkably suppressed proliferative, migratory and invasive functions of melanoma cells.

Recent studies have demonstrated that circRNAs have an important role in disease progression by mediating expressions of their linear transcripts (Wu et al., 2020; Tang et al., 2021; Mecozzi et al., 2021; Peng and Wang, 2021). Through qRT-PCR and rescue experiments, we found that hsa_circ_0062270 was able to stabilize the expression of its linear transcript CDC45. Knockdown of CDC45 blocked proliferative, migratory and invasive functions of melanoma cells, which could be reversed by overexpression of hsa_circ_0062270. The binding of CDC45 to chromatins regulated by the convergent effect of CDKs and DDK coincides with the time point of the start of DNA replication, suggesting that CDC45 is crucial in regulating the initiation of DNA replication (Köhler et al., 2016; Szambowska et al., 2017; Can et al., 2019).

Collectively, our present study was the first attempt to reveal that hsa_circ_0062270 was up-regulated in melanoma specimens and correlated to its prognosis. Hsa_circ_0062270 stimulated malignant process of melanoma by stabilizing its linear transcript CDC45. Our findings provide a new aspect for developing diagnostic and therapeutic strategies for melanoma. Several limitations of our study should be pointed out. First of all, *in vivo* role of hsa_circ_0062270 in melanoma is not explored. Secondly, how hsa_circ_0062270 regulates CDC45 remains unclear. Thirdly, other cell phenotypes of melanoma, including apoptosis, epithelial-mesenchymal transition and cell cycle progression affected by hsa_circ_0062270 are not clear.

## Conclusion

Hsa_circ_0062270 promotes proliferative, migrative and invasive functions in melanoma cells *via* stabilizing the linear transcript CDC45. These findings provided strong evidence that hsa_circ_0062270 could be a novel promising therapeutic target used to diagnosis and treatment of melanoma.

## Data Availability

The original contributions presented in the study are included in the article/Supplementary Material, further inquiries can be directed to the corresponding author.
